# Occurrence of Norovirus and Hepatitis A Virus RNA in Agricultural Soil from Produce Farms in Ohio and Georgia

**DOI:** 10.3390/v18101143

**Published:** 2026-10-15

**Authors:** Efstathia Papafragkou, Michael Kauffman, Gireesh Rajashekara, William Norfolk, Erin Lipp, Qianru Yang

**Affiliations:** 1 Office of Applied Microbiology and Technology, Human Foods Program, Food and Drug Administration, Laurel, MD 20708, USA; yangqr82@gmail.com; 2 Department of Animal Sciences, College of Food, Agricultural, and Environmental Sciences, The Ohio State University, Wooster, OH 44691, USA; mdkauffman.42@gmail.com (M.K.); gireesh2@illinois.edu (G.R.); 3 Department of Environmental Health Science, College of Public Health, University of Georgia, Athens, GA 30602, USA; william.norfolk@uga.edu (W.N.); elipp@uga.edu (E.L.); 4 Division of Biological Sciences, Franklin College of Arts and Sciences, University of Georgia, Athens, GA 30602, USA; 5 Accufy Diagnostics, Rockville, MD 20850, USA

**Keywords:** agricultural soil, human norovirus, hepatitis A virus, foodborne virus, enteric viruses

## Abstract

Although foodborne viruses are considered ubiquitous in the environment, studies on their presence in soil are largely lacking. In this field study, 168 agricultural soil samples were collected from farms in Georgia and Ohio over seven and eight months, respectively. A total RNA isolation method was developed, and extracted genetic material was analyzed using reverse transcription quantitative PCR (RT-qPCR) to detect human norovirus and hepatitis A virus (HAV) RNA. Nearly 6% of samples (*n* = 10) tested positive for HAV RNA, while norovirus genogroup I and II were detected in 1.2% (*n* = 2) and 0.6% (*n* = 1) of samples, respectively. Despite samples being stored at −20 °C for an average of two years prior to testing, viruses remained detectable, although in low levels. CrAssphage DNA and pepper mild mottle virus (PMMoV) RNA were assessed as indicators of human fecal contamination in positive samples; however, neither was detected, precluding any correlation with the viral pathogens—likely due to differing characteristics between the indicators and target viruses. These findings provide valuable insight into the prevalence and diversity of foodborne viruses in agricultural soil and highlight its potential as a vehicle for virus transport to other matrices within a fresh produce farm environment.

## 1. Introduction

Soil harbors a diverse population of microorganisms that can affect human health [1]. While soil pathogens that can directly cause human infections are generally limited, foodborne viruses can be introduced to soil incidentally via contact with contaminated water, or application of biological soil amendments of animal origin (BSAAO) potentially contaminated with human waste. Foodborne viruses are primarily non-enveloped, single-stranded RNA viruses. The viruses most epidemiologically associated with sporadic and outbreak cases of human illness are the human norovirus and hepatitis A virus (HAV) [2]. It has been suggested that foodborne viruses can transfer through soil to fresh produce and subsequently to humans [3]. Though it has never been directly confirmed that soil can be a vehicle for produce contamination, such a route of transmission cannot be completely ruled out. Fresh produce that is likely to be served with little or no additional processing is particularly susceptible to contamination by viruses transmitted via the fecal–oral route.

Once introduced into soil, foodborne viruses’ survival depends on soil physicochemical properties (type, pH level, moisture content, temperature, exposure to sunlight, amount of soluble organic matter), as well as the microbiological quality of soil, and can also vary between viruses and even within strains of the same genogroup [4]. Once contamination occurs, enteric viruses can persist in soil for extended periods, and their association with solids is believed to enhance their persistence. Stability of enteric viruses has been poorly investigated. Previous studies showed that infectious Poliovirus could be recovered from soil for up to eleven days after soil had been irrigated with virus-containing sewage during the summer, while in the winter the virus was detected up to close to three months [5]. Hepatitis A RNA displayed no significant loss when the virus was seeded in different types of animal manure and biosolids and stored at 4 °C for up to sixty days [6].

Though there have been several methods developed and validated for enteric virus isolation and detection in foods, there are very few developed for soil. The limited sample preparation methods that have been published address detection of enteric viruses in sewage, soil or sediment with various recovery efficiencies. Quantification of viruses in such materials via molecular-based methods (primarily PCR) remains problematic due to aggregation of viral particles and inhibitory compounds that naturally exist in such samples. Most of the published literature includes studies targeting the detection of different viruses than those evaluated in this project (i.e., feline calicivirus and murine norovirus as norovirus surrogates), measuring viral infectivity or requiring larger soil quantities (25 g), while the majority of them utilize seeded rather than naturally existing samples.

There is currently limited scientific evidence demonstrating contamination of produce during the growing season through virus transfer from soil, or application of soil amendments, and information on the factors influencing such potential uptake. However, before the risk of virus transmission from soil to crop within a produce-farm environment can be evaluated, it is necessary to first assess the prevalence of viruses of interest in soil. Farm practices, including irrigation, soil amendment types, and worker hygiene, have been highlighted as potential sources of pathogen introduction to the farm environment [7]. Having critical information to compare and evaluate the role of soil characteristics, climate, and farm management practices on pathogen prevalence can significantly improve the understanding of pathogen ecology in agricultural settings.

This study presents an evaluation of virus presence (HAV and human norovirus) and diversity in a variety of different agricultural soil types from two US geographic regions. Our first goal was to develop a method to detect norovirus and HAV RNA levels in different soil types. Soil samples were collected during the 2019 growing season from four farms in Georgia and 14 farms in Ohio. Our second goal was to explore the correlation between the occurrence of human enteric viruses and that of two proposed indicators of human fecal contamination: crAssphage (currently renamed *Carjivirus communis*) and pepper mild mottle virus (PMMoV). The study was unable to continue in 2020 due to restrictions related to the COVID-19 pandemic, which also delayed reporting of these results. To our knowledge this is one of the first reports to provide qualitative data for enteric viruses’ abundance in agricultural soil samples using RT-qPCR in conjunction with the frequency of detection of indicators of human fecal contamination.

## 2. Materials and Methods

### 2.1. Sampling Sites and Sample Collection

Farms were recruited in both Georgia (GA) and Ohio (OH) by voluntary agreement of the farmers. All farms were blinded for the purposes of this study. One hundred and seventeen soil samples from 14 different farms in Ohio and 51 samples from four farms in Georgia were collected by our collaborators at Ohio State University and the University of Georgia, respectively, throughout 2019. In Georgia, soil was collected from four sections per farm, with each section linked to a different crop and, in some cases, management strategy. This study was conducted in parallel with bacterial prevalence studies at the same farms, which have been previously reported [7]. Further details describing the soil collection and shipment are described in that study. Upon receipt, soil samples for viral analysis were stored at −20 °C until processing (up to 24 months). In Ohio, composite samples were made up of three to four random (according to the individual collecting the samples) locations in the field. Three replicate soil samples were collected at each time point, with each replicate sample being a composite of five subsamples from different locations in the field, resulting in approximately 2 kg of soil. All soil samples were kept at temperatures consistent with environmental conditions to minimize changes, then shipped overnight on ice to the FDA facility in Laurel (MD, USA). More information on farm characteristics and metadata collection (soil characteristics [pH, moisture content, composition, and metal ion contents] and climate data [ambient temperature, precipitation]) is presented in our colleagues’ study with data from bacterial pathogen surveillance on the same farms [7].

### 2.2. Method Development and Seeding Studies

A 10% suspension of a norovirus-positive stool sample belonging to genogroup II was utilized for method development from our in-house sample collection [8]. A soil sample from OH, previously tested negative for norovirus, was autoclaved prior to use. For virus spiking, 100 µL of ten-fold serial dilutions of norovirus in phosphate-buffered saline (PBS) was used to seed individual 5 g portions of soil. Experiments were set up in triplicate, and one negative control (no virus added) was also included. Samples were incubated at room temperature to air-dry in a biosafety hood for 10 min to facilitate initial viral adsorption. Virus seeding level ranged from 2 × 10^5^ to 10^2^ RNA copies/g of soil, and recovery was evaluated by RT-qPCR and quantified by an in-house-generated full-length RNA transcript. Limit of detection (LOD) was defined as the lowest viral concentration artificially inoculated (gc/g) that was detected by RT-qPCR. Preliminary efforts to develop a method to isolate virus particles from soil using larger soil quantities (25 g) had poor analytical sensitivity.

### 2.3. Elution of Virus Particles and RNA Isolation from Seeded Soil Samples

Five grams of each soil sample was weighed, in duplicate, into 15 mL conical tubes. Three milliliters of freshly made Tris-Glycine Beef Extract (TGBE, 0.1 M Tris hydrochloride, 0.05 M glycine, and 1% beef extract, pH 9.2) buffer was added to each tube, which was subsequently shaken at 210 rpm at room temperature for 30 min. After a spin at 10,000× *g* at 4 °C for 10 min, the supernatants were filtered through a 0.22 µm pore-size membrane filter with low protein binding (MilliporeSigma, Burlington, MA, USA), mixed with 1 mL Trizol (Thermo Fisher Scientific, Waltham, MA, USA), and incubated at room temperature for 5 min. To each tube, 0.2 mL of chloroform (MilliporeSigma, Burlington, MA, USA) was added, and samples were shaken by hand for 60 s and incubated at room temperature for 2–3 min. After centrifugation at 12,000× *g* at 4 °C for 15 min, the colorless upper aqueous phase was transferred into a new 15 mL tube, mixed with 0.5 mL of isopropanol and incubated at room temperature for 10 min. The RNA pellet was obtained by centrifuging at 12,000× *g* at 4 °C for 10 min, followed by resuspension in 1 mL of 75% ethanol and a subsequent centrifugation at 7500× *g* at 4 °C for 5 min. After removing the supernatant, the RNA pellet was air-dried for 5–10 min in a biosafety II cabinet, then resuspended with either 25 or 80 µL of DNase/RNase-Free water (Invitrogen, Carlsbad, CA, USA). Preliminary supported that increasing the elution volume at this step to 80 µL was necessary so that there is enough nucleic acid for all subsequent assays. The eluted RNA samples were stored at −80 °C until RT-PCR analysis. The focus of this study was to develop a method that yielded high-quality RNA for use in both RT-qPCR-based detection and Whole Genome Sequencing (WGS). This method was not intended to isolate virus particles. Likewise, because this protocol was developed to include specific steps that would generate RNA for WGS (such as inclusion of a filtration step), we did not routinely include a process control virus to document any potential losses during the sample preparation, as it would interfere with identifying sequences of the target viruses that were expected to be in much lower abundance compared to the process control virus.

### 2.4. Detection and Quantification of Viral Genomes with RT-qPCR

Virus detection was performed using the primers and probes for each virus of interest using the RNA isolated from each soil sample. More specifically, the presence of HAV and norovirus RNA copies was quantified using real-time RT-qPCR assays developed on an ABI 7500 Real-Time PCR system (Thermo Fisher Scientific, Waltham, MA, USA) according to protocols developed by the US Center for Disease Control and Prevention for HAV [9] and norovirus [10]. Briefly, the norovirus multiplex real-time assay for GI and GII used the QuantiTect Probe RT-PCR Kit (Qiagen, Germantown, MD, USA) with a 25 μL reaction volume containing 400 nM of each oligonucleotide primer (Cog1F and Cog1R for GI viruses; Cog2F and Cog2R for GII viruses), and 200 nM of each probe (Ring 1E and Ring 2), as well as 100 nM of each primer and probe (ICF, ICR and IACP) for an Internal Amplification Control (IAC). Cycling conditions for amplifying norovirus GI and GII genes included reverse transcription for 30 min at 50 °C, followed by denaturation for 15 min at 95 °C, and then 40 cycles of 95 °C for 15 s and 60 °C for 1 min each. The 25 μL reaction volume for the HAV RT-qPCR assay used the QuantiTect Probe RT-PCR Kit (Qiagen, Germantown, MD, USA) and contained 250 nM forward and reverse oligonucleotide primers, 150 nM probe and 150 nM of probe for IAC (same as for the norovirus assay). The HAV cycling conditions included reverse transcription for 30 min at 50 °C, followed by denaturation for 15 min at 95 °C and then 50 cycles of 94 °C for 15 s, 55 °C for 25 s and 72 °C for 40 s. Serial dilutions of full-length RNA transcripts for norovirus GII produced in our laboratory were used to generate standard curves for RNA quantification (in mean log_10_ genomic RNA copies/g ± standard deviation). The assay could consistently quantify up to 10 genomic copies of GII per reaction.

Sequence Detection Software version 2.3 was used to quantify genomic RNA copies. In both RT-qPCR assays, a non-competitive IAC was directly incorporated into the PCR mix to assess inhibition. A sample was considered positive by RT-qPCR when (1) at least one of the two replicates produced a signal by cycle 40 for norovirus or 50 for HAV, (2) the amplification curve showed a true sigmoidal curve, (3) the positive controls exhibited their expected values, and (4) the negative control was undetected. Inhibition in samples was classified as low, intermediate, or high level if a sample’s IAC had a Ct value within 3 values of the NTC IAC, at least 6 values above that of the NTC IAC, or was not detected, respectively.

### 2.5. Comparison of RNA Purification Kits

Three silt loam soil samples with noticeable RT-qPCR inhibition during screening were selected for evaluation and comparison of two nucleic acid purification kits. The samples had previously tested negative by RT-qPCR for all three viruses (norovirus genogroup I and II and HAV virus). The remaining RNA from the initial 80 µL elution of RNA, after running all PCR assays, was used for further purification following each kit’s manufacturer’s instructions. The kits tested were the Zymo RNA Clean & Concentrator 25 (kit 1) (Zymo, Irvine, CA, USA) and the Qiagen RNeasy^®^ PowerClean^®^ Pro cleanUp kit (kit 2) (Qiagen, Germantown, MD, USA), following manufacturers’ instructions.

### 2.6. Removal of RT-qPCR Inhibition

Once the best nucleic acid purification kit was selected, all 42 soil samples with high levels of inhibition underwent RNA purification and were subsequently re-tested for quantification of norovirus and HAV genomic copies by RT-qPCR. The results were analyzed as previously described.

### 2.7. Detection of Indicators of Human Fecal Contamination in Soil Samples

Soil samples that tested positive for either HAV or norovirus RNA, were re-tested for the presence of PMMoV RNA and crAssphage DNA. For PMMoV detection, a previously published RT-PCR protocol was followed [11]. Briefly, a 25 μL reaction volume using the Qiagen QuantiTect Probe RT-PCR Kit contained 400 nM forward and reverse oligonucleotide primers and 100 nM probe. The cycling conditions included reverse transcription for 30 min at 50 °C, followed by denaturation for 15 min at 95 °C and then 50 cycles of 95 °C for 15 s and 60 °C for 1 min each. As crAssphage is a DNA phage, the sample preparation protocol (described in Section 2.3) was modified by adjusting the Trizol protocol for DNA recovery, according to the manufacturers’ instructions, and omitting the initial filtration step. The PCR protocol for detection and quantification of crAssphage DNA [12] used the Qiagen QuantiTect Probe PCR Kit, 0.6 pmol of each oligonucleotide primer, and 0.3 pmol of FAM-labeled probe. The PCR protocol consisted of an initial denaturation step at 95 °C for 30 s, followed by amplification for 45 cycles at 95 °C for 15 s and 60 °C for 60 s. Quantification was performed with serial dilutions of a 2428 bp segment of the genome, generated and shared with us by the National Calicivirus Laboratory (Atlanta, GA, USA). The limit of detection was 1.7 DNA copies and 0.2 RNA copies per reaction for the crAssphage and PMMoV assays, respectively. We randomly selected twenty samples (five from GA and 15 from OH) that tested negative for either norovirus or HAV and tested them for both indicators.

### 2.8. Data Analysis

Real-time PCR data were initially analyzed and quality-controlled using the ABI 7500 real-time PCR software v2.3 (Applied Biosystems^TM^, Waltham, MA, USA). The viral nucleic acid concentrations for norovirus and HAV were expressed as genome copies (gc)/3 µL of RNA extract and are presented as means and standard deviations determined from at least two independent experiments. The low number of norovirus-positive samples observed (*n* = 3) precluded any statistical analysis. Although the low number of HAV-positive samples (*n* = 10) precluded rigorous statistical modeling, the relationship between the presence of HAV and season, source of irrigation (surface, as in stream or pond, well or non-irrigation), and soil amendment (non-animal, poultry or dairy) was assessed using Fisher’s exact test [13]. The relationship between PCR inhibition level (low, intermediate or high as defined in Section 2.4) and soil amendment and season was assessed for both states and pathogens separately (HAV or norovirus) using Fisher’s exact test. The relationship between inhibition level and irrigation source was further assessed for OH only.

## 3. Results

### 3.1. Optimization of Method Performance

Most commercial RNA isolation kits are only able to process very small amounts (0.2–5 g) of soil, and large sample sizes (25 g) did not provide good detection, so we opted to test two 5 g subsamples to increase the probability of detecting virus RNA. Elution with a glycine buffer (Tris Glycine Beef Extract, TGBE, pH 9.2) that also contains beef extract has been used extensively in the extraction of viruses from foods but also from several types of sewage sludge [14]. The presence of beef extract in the elution buffer was expected to help the desorption of virus from soil particles [15]. Although using larger volumes of elution buffer is expected to more efficiently recover virus particles, we opted for a very small volume of elution buffer, as we refrained from using any concentration steps. Similarly, the use of the TRIzol^®^ reagent has been widely reported for virus RNA isolation from a variety of matrices with high content of PCR inhibitors such as plant tissues. The TRIzol^®^ reagent was selected as it can be adapted to different starting volumes and can be used to isolate either RNA (i.e., norovirus) or DNA targets (i.e., crAssphage). One of the advantages of having an overall short sample preparation is that virus RNA is less likely to be degraded or lost during this procedure. The recovery of the newly established isolation method ranged from 0.05 to 0.07%, and seeded norovirus GII could be consistently detected at levels as low as 10 RNA copies/5 g sample when 3 µL of RNA template was added to a final reaction volume of 22 µL (Table 1). The lack of a standardized method for recovery of viruses from soil necessitates the inclusion of a process control virus spiked before all processing steps to measure the performance and variability per sample of the methodology. The RNA copy limit of detection established during method development reflects assay sensitivity under idealized conditions and cannot be extrapolated to field sample performance. Accordingly, as Ct values were not converted to viral concentrations, results from the field samples are reported as presence/absence of target viral nucleic acid.

### 3.2. RNA Purification

A comparison of the inhibition observed in the two RT-qPCR protocols found that 19% (*n* = 32) of the samples showed inhibition using the norovirus RT-PCR protocol, while only 5.95% (*n* = 10) showed inhibition with the HAV protocol. Dilution of the RNA template prior to RT-qPCR did not consistently improve detection). As diluting inhibitors can also dilute the target RNA, especially when expected in low quantities, an RNA purification step prior to RT-qPCR was evaluated. Zymo RNA Clean & Concentrator (kit 1) and Qiagen RNeasy^®^ PowerClean^®^ Pro Cleanup (kit 2) were selected and used according to the manufacturers’ instructions. Both purification kits were able to significantly remove most inhibitory substances in two out of three soil samples, as indicated by their IAC Ct values (Table 2). However, inhibition could not be effectively removed in the third sample that had slightly higher organic matter (OM) and sand content, as well as slightly lower silt and clay concentration compared to the other two (negative numbers indicate no improvement in inhibition). Similar differences in results after clean-up were observed for both viruses analyzed. Although overall the yield between the two kits was comparable for both RT-PCR protocols, the Zymo RNA Clean & Concentrator (kit 1) was chosen to clean all soil samples exhibiting high PCR inhibition as it was simpler and faster to use.

### 3.3. Soil Metadata and Virus Detection

In OH, 117 samples were collected from 14 farms, while in GA, 51 samples were collected from four fields throughout the calendar year of 2019 (Table 3). Samples were collected from OH from February to November and from GA from March to October. More samples were tested primarily during the summer months in both states, as this was the growing season for all planted crops.. A more detailed description of the metadata, including metal concentrations, is presented in Table S1 in the Supplementary Materials and also referenced in an earlier publication [7]. From a total of 168 soil samples tested by RT-qPCR for the presence of HAV and norovirus (genogroups GI and GII) RNA, 13 samples were positive for the presence of one of the viruses (7.7% of total samples analyzed) (Table 3). Among the 117 samples collected from OH, six samples were PCR-positive for HAV (5.1%) with cycle threshold (Ct) values ranging from ~38 to 42. Similarly, from the 51 samples originating from GA, four samples tested positive for HAV (7.8%) with Ct values > 35. In OH, HAV was only detected in farms from June to September, while in GA it was detected in June, July, and then again in September, suggesting the possibility of an intervening contamination event or that fluctuations in weather during August could have affected viral stability. However, given the limited number of positive samples, this is a conjecture at this time. Norovirus was detected in only three samples in OH (2.6%), with two of these belonging to genogroup I and one to II, with Ct values close to 40. All 13 samples that exhibited a PCR-positive signal for either of the viruses were collected between June and October (Table 3). No co-contamination was observed in any of the samples evaluated. In GA, 87% of the samples and 81% of the samples in OH were gathered in the summer (June, July, and August) and fall (September and October), which also corresponds to the seasons with the highest observed HAV prevalence. Although this imbalance might imply a relation between frequency of sampling and HAV prevalence, the number of samples is accounted for (i.e., adjusted for) in the Fisher contingency table. Although Fisher’s exact test is valid for small samples, a sample size of *n* = 10 represents a genuine constraint, carrying with it the inherent limitation of reduced statistical power.

No relationship was observed between season (*p* = 1 in OH and GA, Not Significant) and HAV detection, or between soil amendment (*p* = 0.6 in OH and *p* = 0.62 in GA, N.S.) and HAV detection (Figures S1 and S2). In OH, irrigation appears to have a modest effect (*p*-value = 0.07) on HAV prevalence, with non-irrigated and surface (stream or pond)-irrigated fields having a higher rate of virus detection (Figure S2). As all GA samples were well-irrigated, the impact of irrigation on HAV prevalence could not be evaluated. This observation is in agreement with other studies referencing that among different water matrices, HAV was more prevalent in surface water rather than groundwater [16,17]. Overall, as limited samples tested positive for the viral targets, no direct association between certain soil physicochemical properties and management practices and virus presence could be investigated.

### 3.4. Evaluating and Removing RT-qPCR Inhibition in Soil

RT-qPCR inhibition in RNA samples was assessed (Section 2.6), and scoring of RT-qPCR inhibition for both viruses is presented in Table 4. For soil sampled from farms in OH, the majority had low or intermediate inhibition (~80%), while high RT-qPCR inhibition was observed in around 20% of samples when screened for norovirus. A similar trend was recorded for samples from the same state analyzed for HAV presence through RT-qPCR; the overwhelming majority (95%) had low or intermediate RT-qPCR inhibition. Likewise, in GA, high inhibition was observed in only 16% of the samples using the norovirus RT-qPCR assay and close to 8% with the HAV RT-PCR protocol. In Ohio, the distribution of HAV and norovirus inhibition levels differed significantly by soil amendment type (P_HAV_ = 0.003, P_Norovirus_ = 0.02). Namely, poultry-amended fields had the highest proportion of low HAV and norovirus inhibition, while dairy- and non-amended fields showed slightly higher proportions of intermediate and high inhibition. In Ohio, season was significantly associated with norovirus inhibition levels (*p* = 0.028), with summer samples showing higher proportions of intermediate and high norovirus inhibition (Figures S1 and S2). As these are just observations from this study, the effect of RT-qPCR inhibition and metadata of each farming practice can be studied further in a larger or more controlled study to truly ascertain more defined relationships.

For 42 samples presenting high RT-qPCR inhibition in the initial screening, an RNA purification step was performed after the TRIzol^®^ extraction using a Zymo RNA clean & concentrator kit. Notably, more than 96% (41 out of 42) of those samples demonstrated a lower level of inhibition when re-tested with individual virus RT-PCR assays, indicating that sequential treatment was effective in improving viral detection in most of the samples. None of the samples initially scored as negative turned out to have an RT-PCR signal, demonstrating that the negative result was not attributable to PCR inhibition.

### 3.5. Screening for Indicators of Human Fecal Contamination

When the 13 positive (HAV or norovirus) samples were screened for the presence of either PMMoV or crAssphage, none tested positive for these proposed indicators. Additionally, twenty samples, negative for HAV/norovirus, were randomly selected and tested for the same indicators, and none of them displayed positive PCR signals.

## 4. Discussion

To date, a number of different studies have assessed the prevalence of foodborne viruses in various different settings and matrices ranging from foods, surfaces, biosolids, raw sewage, agricultural water and wastewater, highlighting how widespread viral contamination can be in the environment. In this study, our goal was to develop a method and screen a large number of soil samples from farms in two states (Ohio and Georgia) with different soil characteristics to study the relative abundance and diversity of HAV and norovirus RNA load throughout the year. Roughly 5% and 8% of the samples, in OH and GA respectively, tested positive for the presence of HAV RNA. Norovirus RNA was detected in less than 3% of samples in OH and in none of the GA samples. Notably, the majority of HAV-positive samples in both GA (3/4) and OH (4/6) were detected in the summer months, and the same seasonal trend was true for norovirus in OH, as the testing rate was higher during those months for both locations. Conversely, when samples from the same farms were tested for bacteria Salmonella, Shiga toxin-producing *Escherichia coli*, and Campylobacter, they were detected at low rates in soil and water (less than 10%), while Listeria monocytogenes was found in 13% and 18% in soil and water respectively [7]. HAV was more frequently detected than norovirus, which is expected as HAV is notoriously recognized as environmentally stable and, at the time of the study, on the rise in several states in the U.S [18,19]. According to CDC surveillance data for Ohio in 2019, an increase in reported norovirus outbreaks was observed, though a direct association with the findings of this study cannot be established [20]. Year-round sampling would have yielded a more comprehensive understanding of viral dynamics, as demonstrated by a recent study in which HAV was detected more frequently than norovirus over a sixteen-month wastewater surveillance period [21]. Integrating wastewater surveillance in conjunction with soil testing offers a more comprehensive picture of enteric viruses’ prevalence and transmission dynamics in a farm setting and could be included in future studies.

Published studies on enteric viruses’ distribution and persistence in agricultural soil remain limited. An earlier study conducted in soil in the vicinity of the Mutha River in India showed that among 403 samples collected, HAV and Hepatitis E virus were detected in 19.1% and 4.9% of samples respectively [22]. Persistence of fecally derived viruses in coastal and estuarine sediments can vary depending on temperature and viral species, with poliovirus remaining stable for more than a month in estuarine sediment stored at 4 °C [23]. A more recent study conducted in South Korea investigating the occurrence of enteric viruses in fresh produce and its production environment detected no norovirus or HAV in one hundred ninety-one soil samples tested [24]. In our study, viral loads were detected primarily in the warmer months, as the collection of the majority of samples tested originated from that time. Although there has been some earlier evidence that norovirus from genogroup I is more environmentally stable than genogroup II [25], the very low positivity rate for both genogroups in this study and the fact that the majority of samples tested originated during the warmer months did not allow for any significant seasonal trends to be observed.

Foodborne viruses are primarily single-stranded RNA, non-enveloped viruses and can be isolated from contaminated matrices with methods that target either extraction of viral particles (indirect methods) or total nucleic acids (direct methods). There is not currently a broadly available cell culture method for either wild-type HAV or norovirus; therefore, molecular methods, and primarily reverse transcription-PCR (RT-PCR), are the most widely used assays for detection. Similar to our study, testing small quantities of sample and applying total RNA isolation has been applied to other matrices where viral contamination is not expected to be on the item surface, and RT-qPCR inhibition is likely (i.e., deli meats, sausage, liver) and, in such conditions, low recoveries are not unexpected [26,27]. Earlier studies on the presence of enteric viruses in sludge or sewage cannot be directly compared with this study, as soil has different properties compared to these matrices. The recovery rate using TRIzol^®^ reported by Miura et al. [28] is comparably low (0.10%) to the one presented in this study, although those authors quantified recovery from sediments using poliovirus as a model virus. Many of the studies investigating recovery of viruses/phages from biosolids and sediments have used RT-PCR-based detection assays. Other studies using poliovirus, coxsackievirus, and echovirus in different types of sediment (estuarine, freshwater and marine) have developed elution/concentration methods that measure infectious virus recovery [29,30], while there is only one study recovering infectious HAV from freshwater sediment [31]. Several of the methods have been developed using artificial inoculation of the virus onto the matrix, which is different than testing a method with naturally existing samples. Recoveries of viral RNA from sediments range from 0.09% to 11% for direct extraction and RT-qPCR [14]. The recovery percentages for infectious Murine norovirus in three types of agricultural soils were very similar to each other (54.3%, 54.4%, and 56.9%). In contrast, the titers of infectious Feline Calicivirus varied significantly depending on the type of soil, with 47.8%, 15.6%, and 17.7% recovered from black, loamy, and sandy soil, respectively [32].

As witnessed widely in our study, such a considerably heterogeneous matrix like soil can have a number of compounds that can compromise molecular detection, largely humic and fulvic acids that are mainly produced by the organic matter biodegradation [33,34]. Soil composition can vary by several parameters, including location, irrigation and physicochemical properties, which further impact the efficiency of PCR-based amplification. Sandy soil has been shown to have more PCR inhibitors extracted compared to loamy and black earth soil. An earlier study by Prado and colleagues investigating recovery rates of human adenovirus, rotavirus, norovirus and HAV from sewage sludge identified a prevalence of less than 7.5%, demonstrating that the highly concentrated inhibitor level can affect PCR performance [35]. We chose to compare two commercially available RNA purification kits that have been previously shown to efficiently remove RT-qPCR inhibitors with three samples of silty soil. Our data showed that after isolating total RNA with the TRIzol^®^ reagent, both kits used removed inhibition in two out of three samples tested, though not all interfering components were completely removed. To exclude the likelihood that low virus concentrations detected were not attributable to RT-qPCR inhibition, an RNA purification step was introduced prior to RT-qPCR. When 32 RNA samples that showed high RT-qPCR inhibition were purified and re-tested, none of them came out positive, confirming the effective removal of inhibitors and the absence of any virus RT-qPCR signal.

The prototypic CrAssphage (p-crAssphage) is a double-stranded DNA phage that was discovered almost a decade ago and since then has been extensively investigated as an indicator of human fecal pollution in water environments due to its abundance and strong correlation with enteric viruses [12,36,37]. Similarly, PMMoV has been reported as a suitable indicator of human fecal contamination for monitoring water quality in drinking water, river water, groundwater and wastewater [38]. Although crAssphage and PMMoV have been characterized as the most abundant DNA and RNA virus respectively in human feces, none of the soil samples that tested positive for the presence of norovirus or HAV tested positive for either of the human fecal indicators. In the limited sampling of soils that no virus was detected (twenty in total), no indicators could be recovered either. The lack of co-occurrence could be attributed to a number of reasons, including different recovery efficiencies and stability characteristics due to different capsid structure and morphology. When these indicators are detected in samples that have been tested positive for enteric viruses, they are commonly found in higher concentrations than the viruses [39,40]. In this study, the lack of correlation could be explained by the absence of a concentration step during sample preparation. Increasing the sample size would have necessitated a concentration step that would have likely increased the method’s sensitivity as well as the possibility of detecting both viruses and indicators. However, analyzing larger sample sizes may carry a proportionally higher burden of inhibitory compounds co-purified alongside viral nucleic acids. Individual indicators may also associate more effectively with certain viruses under each environmental or geographical setting. Notably, most of the work published on the association between enteric viruses and indicators has targeted norovirus and less frequently HAV [39,41,42]. Lastly, correlation between viruses and indicators may vary as their individual fate, particle association and transport upon release in the environment may differ [43].

There is currently limited information on the occurrence and stability of these indicators in soil. CrAssphage has been detected in estuarine sediments in Australia in a concentration of 1.9 × 10^4^ gc/g and in 74% of surface soil samples from vegetable farms in China [44]. It could also be quantified in sewage-contaminated sandy sediment in an outdoor mesocosm for up to 40 days [11]. However, in a few studies, crAssphage concentration does not always positively correlate with enteric viruses’ presence as evidenced in surface water, sediments and process water [39,45]. Respectively for PMMoV’s covariation, significant correlation between this indicator and enteric viruses (Aichi virus, HAV, and Norovirus GI and GII) has been demonstrated in Mid-Atlantic surface and reclaimed waters but not in surface water samples [46]. In a study looking into the decay rate of norovirus GII, crAssphage and PMMoV in both a laboratory-controlled and an in situ estuarine setting, crAssphage decayed marginally faster in the laboratory setting and sunlight treatment consistently resulted in enhanced inactivation in both settings [41]. The negative correlation observed in this study is probably related to the low number of positive samples and low viral contamination level. Further investigation is needed on the co-abundance and distribution of enteric viruses and indicators in settings other than solely aquatic to establish a more reliable association between them.

This study is subject to several limitations. First, the absence of localized human prevalence data prevents the determination of any definitive link to the contaminated soil samples. As for the methodological limitations, a sample preparation method was developed using only one norovirus genogroup as the model virus, which may not accurately reflect the performance of the method for HAV or the indicator organisms. It also cannot be ruled out that storing the soil samples for a prolonged time at −20 °C prior to testing might have introduced an underestimation of the viral titer. This study prioritized sample collection during the warmer months, when heightened human activity on farms is known to increase the risk of virus introduction and transmission within the farm environment. As a consequence, the prevalence rates reported here reflect conditions specific to this period and should not be interpreted as representative of year-round distribution. As spiking of samples with a process control virus was omitted, results are reported as presence/absence only, with negative results interpreted as “not detected” rather than confirmed absence. These limitations preclude quantitative interpretation of the surveillance data, and observed differences in detection frequency across sites or time points should be interpreted with caution, as they may reflect variability in method performance rather than true differences in viral occurrence. Future iterations of this method should incorporate a validated process control virus spiked prior to all processing steps (i.e., Mengovirus, Murine Norovirus), in accordance with ISO 15216 [47]. Additionally, during storage, no action was taken to reduce virus aggregation or prevent moisture evaporation, which may have affected virus recovery over time. As the virus load was low, no confirmation through WGS could be performed. The low positivity rate also precluded any specific correlations between soil properties and practices that may affect virus presence or persistence. Lastly, as viruses are typically not distributed evenly and are in low concentrations, the selection of subsamples tested may partially explain the low recovery observed.

## 5. Conclusions

Limited work has been published monitoring the distribution of enteric viruses in soil. A fast method was developed for detection of HAV and norovirus RNA in agricultural soil, which was first evaluated in a limited number of artificially contaminated samples and then applied to naturally occurring soil samples. Due to the inhibition observed in a significant number of samples analyzed, a clean-up step was incorporated in the sample preparation protocol. This total RNA isolation method can be used for screening for the presence of several viruses circulating in soil. Using this method on samples that have been archived for at least two years at −20 °C, low HAV and norovirus loads and few positive samples were detected in the soil from two states with different types of farm soil in the United States. The sporadic detection of the two viruses did not correlate with the presence of crAssphage and PMMoV, selected indicators of human fecal contamination. The lack of association between fecal indicators and virus presence underscores the failure of a single indicator, suggesting that indicator suitability may differ for each setting/virus. Future work can build on this study by improving the method for field testing of “fresh” soil to monitor virus abundance throughout the year, as well as for experimental longitudinal studies in controlled conditions in growth chambers where viruses’ survival in variable environmental conditions can be systematically evaluated.

## Figures and Tables

**Table 1:** Recovery of seeded norovirus (genogroup GII) from agricultural soil.

Seeded (Avg Ct Value)	Seeded (Avg RNA Copies)	Detected (Avg Ct Values)	Detected (Avg RNA Copies)	#+ Replicates	RT-qPCR Inhibition
16.55 ± 0.07	(3.02 ± 0.14) × 10^6^	27.36 ± 0.25	(1.67 ± 0.29) × 10^3^	3/3	Low
19.39 ± 0.07	(4.72 ± 0.23) × 10^5^	30.51 ± 1.12	(3.32 ± 2.45) × 10^2^	3/3	Low
22.73 ± 0.10	(5.27 ± 0.34) × 10^4^	34.68 ± 0.42	(2.62 ± 0.65) × 10^1^	3/3	Low
26.16 ± 0.06	(5.54 ± 0.20) × 10^3^	39.15 ± 2.51	3.34 ± 2.74	2/3	Low

**Table 2:** Comparison of RNA purification kits for eliminating RT-qPCR inhibitors from agricultural soil collected from farms.

Sample ID	Texture	pH	% OM	% Sand	% Silt	% Clay	HAV ΔIAC Ct Values		Norovirus ΔIAC Ct Values	
							Kit 1	Kit 2	Kit 1	Kit 2
1	Silt loam	7.2	3.6	23.6	58.2	18.1	3.7	3.6	4.5	4.2
2	Silt loam	5.7	2.6	22.5	58.1	19.4	2.5	2.5	2.3	2.1
3	Silt loam	6.5	4.4	27.2	55.9	16.9	−0.2	1	−0.5	−1.9

**Table 3:** Overall prevalence of norovirus and hepatitis A virus in agricultural soil from Ohio and Georgia, US by state and month.

Regions	Seasons	Number of Samples Analyzed	Number of Norovirus-GI Positive Samples (%)	Number of Norovirus-GII Positive Samples (%)	Number of HAV Positive Samples (%)
GA	March–May	7	0 (0%)	0 (0%)	0 (0%)
	June–Aug	33	0 (0%)	0 (0%)	3 (9%)
	Sep–Nov	11	0 (0%)	0 (0%)	1 (9%)
	Dec–Feb	0	0 (0%)	0 (0%)	0 (0%)
OH	March–May	13	0 (0%)	0 (0%)	0 (0%)
	June–Aug	60	1 (1.6%)	1 (1.60%)	4 (6.6%)
	Sep–Nov	35	1 (1.6%)	0 (0%)	2 (5.7%)
	Dec–Feb	9	0 (0%)	0 (0%)	0 (0%)
Total		168	2 (1.2%)	1 (0.6%)	10 (5.96%)

**Table 4:** RT-qPCR inhibition level in RNA samples from soil and change in inhibition after RNA purification with Zymo RNA Clean & Concentrator kit.

Target Virus	Collection Site	Inhibition Level BEFORE Purification	Count	Inhibition Level AFTER Purification	Count
Norovirus	OH	Low	88	Low	102
		Intermediate	5	Intermediate	14
		**High**	**24**	**High**	**1**
	GA	Low	34	Low	40
		Intermediate	9	Intermediate	11
		**High**	**8**	**High**	**0**
Hepatitis A virus	OH	Low	99	Low	101
		Intermediate	12	Intermediate	16
		**High**	**6**	**High**	**0**
	GA	Low	34	Low	37
		Intermediate	13	Intermediate	14
		**High**	**4**	**High**	**0**

## Data Availability

The original data presented in this study are included in the article/Supplementary Material. Further inquiries can be directed to the corresponding author.

## Supplementary Materials

The following supporting information can be downloaded at: https://www.mdpi.com/article/10.3390/v18101143/s1, Supplementary Table S1. Results (RT-qPCR) and metadata associated with soil samples evaluated in this study. Farm subfield ID indicates the division of samples within the GA fields according to produce type (not indicated). Soil Amendment Type: P for poultry sources, D for dairy source, C for composted, If no D or P is indicated the BSA was green compost. Composted indicates whether an amendment was composted (1) or not (0) where known. Rotation, Y indicates a farm in rotation while N is in production. Supplementary Figure S1. RT-qPCR Inhibition Profiles of Hepatitis A Virus Across Irrigation Methods (Irrig), Soil Amendment Types (SA), and Season for GA and OH. Supplementary Figure S2. RT-qPCR Inhibition Profiles of Norovirus Across Irrigation Methods (Irrig), Soil Amendment Types (SA), and Season for GA and OH.
