# Supplementary material for: Occurrence of Norovirus and Hepatitis A Virus RNA in Agricultural Soil from Produce Farms in Ohio and Georgia
Source: Viruses. 2026 Oct 15;18(10):1143. doi: 10.3390/v18101143 (PMC13709443; doi:10.3390/v18101143)
Supplement: Supplementary file 1 [file viruses-18-01143-s001.zip › viruses-4437835-Supplemental Figures 5.10.2026.pdf]

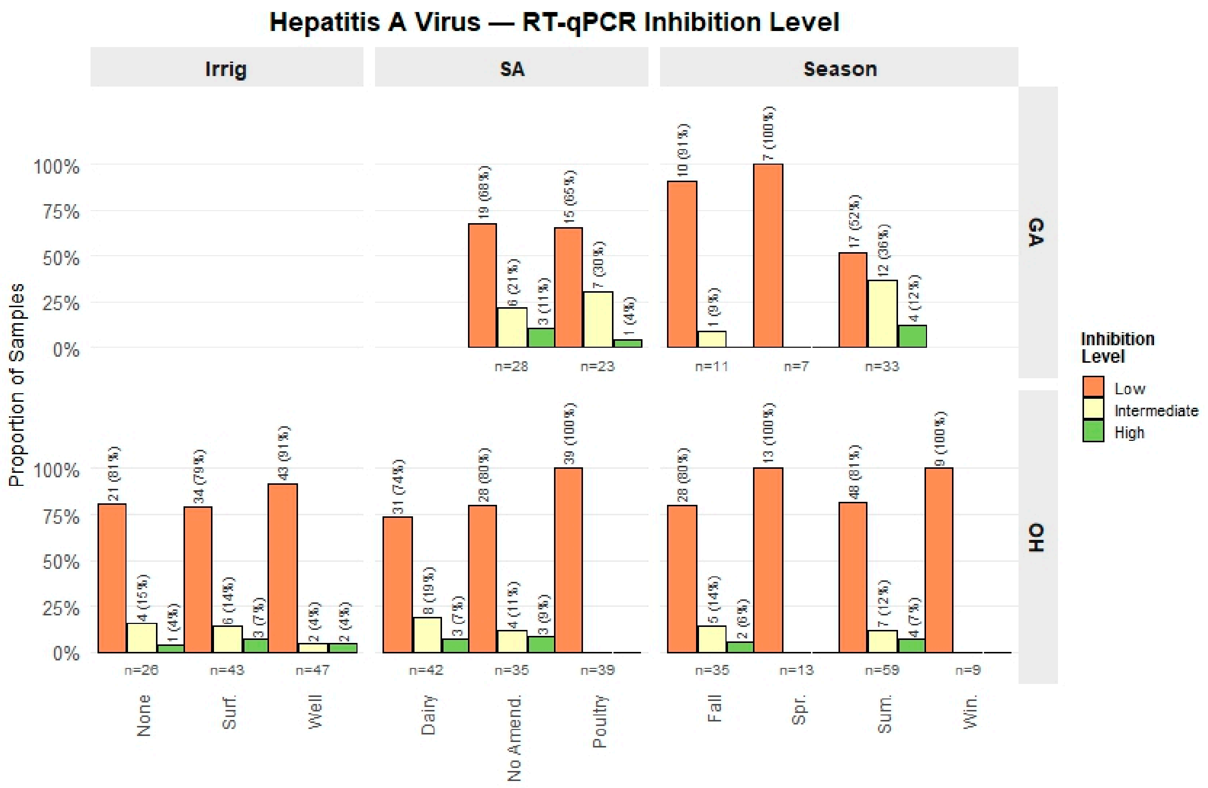

Figure S1: RT-qPCR Inhibition Profiles of Hepatitis A Virus Across Irrigation Methods (Irrig), Soil Amendment Types (SA), and Season for GA and OH.

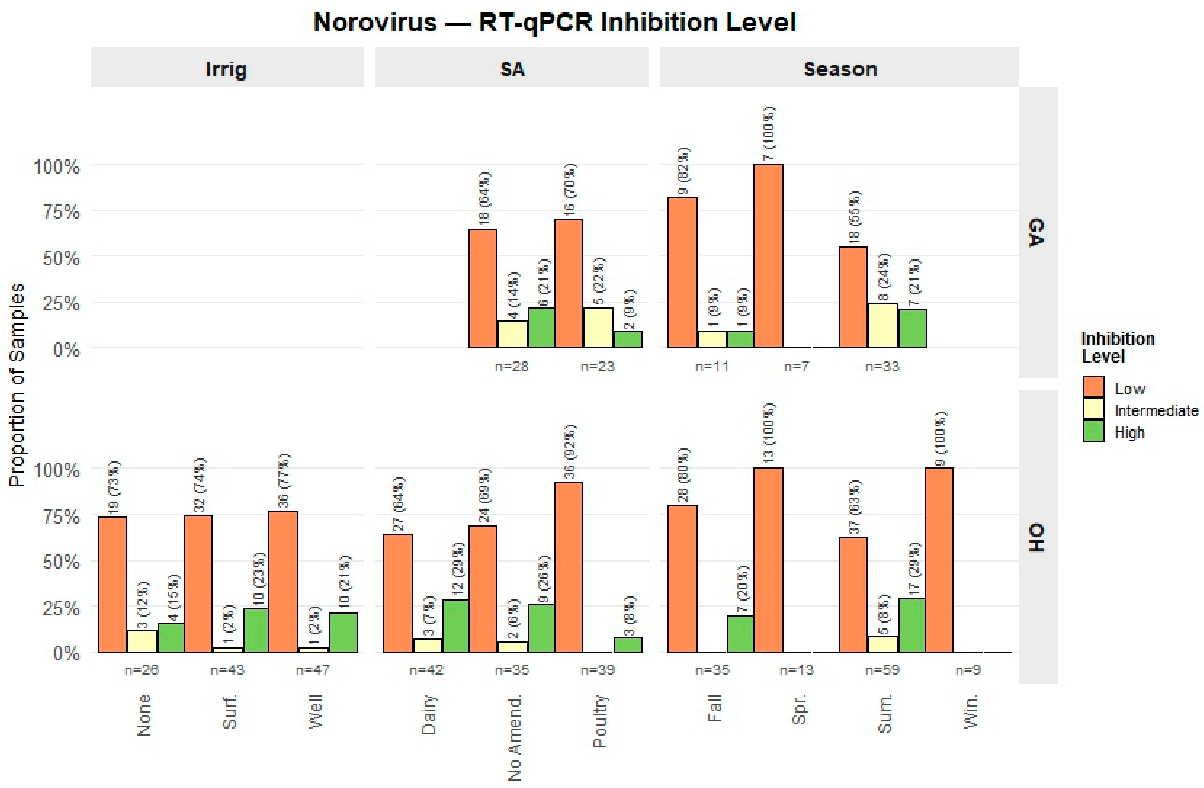

Figure S2: RT-qPCR Inhibition Profiles of Norovirus Across Irrigation Methods (Irrig), Soil Amendment Types (SA), and Season for GA and OH.
